# Assessment of Central Sensitization in Breast Cancer Survivors: Convergent Validity and Use of the Central Sensitization Inventory (CSI) and Its Short-Form as a Clustering Tool

**DOI:** 10.3390/clinpract11030076

**Published:** 2021-09-07

**Authors:** Alban Hurth, Jessica Nijzink-Ter Steege, Pauline Scheepbouwer, Eva Roose, Astrid Lahousse, Laurence Leysen, Lara Stas, Jeroen Kregel, Eric Salvat, Jo Nijs

**Affiliations:** 1 Pain in Motion Research Group (PAIN), Department of Physiotherapy, Human Physiology and Anatomy, Faculty of Physical Education & Physiotherapy, Vrije Universiteit Brussel, 1050 Brussel, Belgium; jessicanijzink@hotmail.com (J.N.-T.S.); pauline_1987@hotmail.com (P.S.); Eva.Charlotte.S.Roose@vub.be (E.R.); astrid.lucie.lahousse@vub.be (A.L.); Laurence.Leysen@vub.be (L.L.); 2 Institut de Formation en Masso-Kinésithérapie de Strasbourg, Université de Strasbourg, 67000 Strasbourg, France; 3 Interfaculty Center for Data Processing and Statistics, Core Facility, Vrije Universiteit Brussel, 1050 Brussel, Belgium; Lara.stas@vub.be; 4 Biostatistics and Medical Informatics Research Group, Faculty of Medicine and Pharmacy, Department of Public Health, Vrije Universiteit Brussel, 1050 Brussel, Belgium; 5 Breederode Hogeschool, 3011 Rotterdam, The Netherlands; jeroenkregel@gmail.com; 6 Centre d’Evaluation et de Traitement de la Douleur, Hôpitaux Universitaires de Strasbourg, 67000 Strasbourg, France; eric.salvat@chru-strasbourg.fr; 7 Centre National de la Recherche Scientifique, Institut des Neurosciences Cellulaires et Intégratives, 67000 Strasbourg, France; 8 Chronic Pain Rehabilitation, Department of Physical Medicine and Physiotherapy, University Hospital Brussels, 1090 Jette, Belgium; 9 Unit of Physiotherapy, Department of Health and Rehabilitation, Institute of Neuroscience and Physiology, Sahlgrenska Academy, University of Gothenburg, 40530 Gothenburg, Sweden; 10 Center for Person-Centred Care (GPCC), Sahlgrenska Academy, University of Gothenburg, 40530 Gothenburg, Sweden

**Keywords:** breast cancer, cancer survivors, chronic pain, central sensitization, central sensitivity syndrome, central sensitization inventory

## Abstract

The Central Sensitization Inventory (CSI) measurement properties in patients having nonspecific, noncancer pain are well-established. However, studies examining the reliability and validity of either the CSI or the Central Sensitization Inventory short-form version (CSI-9) in breast cancer survivors (BCS) are scarce. The purpose was to evaluate convergent validity and internal consistency of the CSI and CSI-9. Additionally, the relevance of a new cluster calculator using the CSI was explored. The cross-sectional multi-center study included 65 BCS and 37 healthy volunteers. Patients filled out multiple questionnaires assessing pain, number of painful areas, anxiety, depression and quality of life. The relevance of a cluster calculator was explored by known-group comparisons and boxplot description. All hypotheses were formulated before data analysis. The majority of hypotheses on the correlations between the CSI or CSI-9 and other health outcomes were confirmed (22 out of 27). The CSI and CSI-9 have excellent (α = 0.92) and good (α = 0.86) internal consistency, respectively. The CSI cluster calculator might be an interesting tool to use to have a patient’s overall condition snapshot. Generally, the study findings support the construct validity and internal consistency of the CSI, which underline the use of this self-reported instrument in BCS. The CSI-9 shows promising results, but should be further evaluated.

## 1. Introduction

The Global Cancer Observatory estimated that around 2.3 million new female breast cancer cases were diagnosed worldwide in 2020 [1]. Among women, breast cancer is the most commonly diagnosed cancer, accounting for almost 1 in 4 cancer cases, and the leading cause of cancer death [1]. An individual is considered a cancer survivor from the time of diagnosis, through the balance of his or her life [2]. Advances in treatments and early detection, as well as ageing of the global population, have led to growing numbers of breast cancer survivors (BCS). Furthermore, pharmacotherapy-based treatment in breast cancer using different drugs has gained attention and acquired progression [3]. For those diagnosed in the period 2008–2014, the 5-year relative survival rate is above 90%; therefore, they might face long-term consequences of cancer and its related treatments, even beyond treatment completion [4]. The cancer survivorship burden is not restricted to physical symptoms, but also affects social life, psychological state, employment and overall quality of life [5].

Among these consequences, pain is a frequently occurring symptom in cancer survivors, with a prevalence higher than 20% after curative treatment [6]. Pain is an unpleasant experience drawing on a large array of information, including but not limited to nociception, beliefs, past experiences, psychological state and social environment. Pain can be classified based on its underlying mechanism as nociceptive, neuropathic or nociplastic. The neurophysiological mechanisms thought to underlie nociplastic pain are regrouped under the term central sensitization (CS), which is defined as an “increased responsiveness of nociceptive neurons in the central nervous system to their normal or subthreshold afferent input” [7]. This broad term encompasses various changes at several levels of the central nervous system such as: long term potentiation, and dysregulation in pro-nociceptive and anti-nociceptive pathways [8,9]. At least a subset of BCS displays signs and symptoms indicative of nociplastic pain [10]. In clinical practice, pain arising from CS is classically widespread and characterized by allodynia and hyperalgesia [11]. Besides pain, other symptoms are often co-occurring including fatigue, mood disturbances, concentration and memories difficulties. Additional challenges come with this kind of clinical picture; therefore, it is of primary importance to assess them appropriately to further guide treatment. A gold standard to detect CS is still lacking; however, guidelines have been published to help clinicians in their assessment and treatment of BCS [11,12].

The Central Sensitization Inventory (CSI) was initially designed as a self-report measure to assess CS-related symptoms severity and screen for the presence of Central Sensitivity Syndromes (CSS) [13]. The rationale for CSS was that many syndromes considered as unexplained like fibromyalgia or irritable bowel syndrome shared a general hypersensitivity to various stimuli and a range of symptoms. It was therefore hypothesized that CSS share a common etiology of CS [14]. Recently, a shorter version of the CSI called the CSI-9 has been developed [15]. The main goal of this abbreviated tool is to facilitate a quick screening and reduce the participant’s burden, while keeping acceptable measurement properties. In addition, a cut-off score and severity levels have been proposed to facilitate the interpretation of the CSI [16,17,18]. Recently, Cuestas Vargas et al. developed a CSI cluster calculator (CSI-CC) that proposes to classify patients in three groups of CS-related symptom severity based on their answers [19]. The psychometric properties of the CSI in patients having nonspecific, noncancer pain are well-established, but studies examining the reliability and validity of either the CSI or the CSI-9 in cancer patients or cancer survivors are scarce [20,21,22]. This is an important knowledge gap, especially given the importance of CS as a key underlying mechanism for pain in BCS, as well as the potential of the CSI to serve pain phenotyping in cancer survivors [10,11,23].

### Study Objectives and Hypotheses

The aims of the present study in BCS were to evaluate the internal consistency of both questionnaires and evaluate their convergent validity with related health outcomes in BCS. A secondary aim was to investigate how the three subgroups formed with the CSI-CC differ on various health outcomes.

Prior to the analysis, we hypothesized that an excellent and a good internal consistency would be found, respectively, for the CSI and CSI-9 in BCS. Given its factor structure and items, it was hypothesized that the CSI scores would show a very strong correlation with the CSI-9; a moderate correlation with the pain Visual Analogue Scale (VAS), Widespread Pain Index (WPI), Hospital Anxiety and Depression Scale (HADS) anxiety, HADS depression and the European Organization for Research and Treatment of Cancer Quality of Life-Questionnaire C30 (EORTC) physical, role, emotional, cognitive, fatigue, pain, insomnia and global health status; and a weak correlation with the EORTC social. The CSI-9 may yield correlation coefficients lower than the original CSI, but the reduction should not drop by more than 0.10 from the CSI [15]. Finally, it was hypothesized that a progressive worsening in the different outcome measures from the lower to the higher severity clusters will be observed.

## 2. Methods

The COSMIN consensus on health-related patient reported outcome was used as guidelines and used to report this study [24,25].

### 2.1. Participants and Procedure

BCS were consecutively recruited by independent physiotherapists and physiotherapists from our research team (J.NTS and P.S) at several private practices in the Netherlands. Female healthy volunteers were conveniently recruited by approaching people from the general population in the environment of the researchers (colleagues, friends, family). All participants and participating physical therapists were unpaid volunteers.

BCS were included if they were: native Dutch speakers; had been affected by a breast cancer diagnosis with or without pain related to their cancer or cancer treatment. Patients were excluded if: they were still receiving any type of cancer treatment except for hormone therapy or immunotherapy in the last 30 days before assessment, or they had metastases in their medical history, or pain was unrelated to cancer or cancer treatment. All data that were collected were used to assess if the pain was related to cancer or cancer treatment.

Healthy volunteers were included if they were: native Dutch speakers, were not experiencing any pain and were not diagnosed with any (chronic) disease at the time of their assessment. The female healthy volunteers were only used for the CS-related symptoms subgroups analysis. All participants were provided with study information and informed consent was obtained prior to study enrollment.

### 2.2. Measures

Each physical therapist that participated in the study received a protocol for administering the questionnaires and informational packages intended for participants consisting of: written information regarding the research protocol, an informed consent sheet, a social demographic questionnaire and the questionnaires of interest in a random order.

BCS were asked to fill out the following questionnaires in their Dutch version: the CSI, VAS, WPI, HADS, EORTC and a social demographic questionnaire. The CSI-9 was extracted from the full CSI. The same questionnaires were administered to female healthy volunteers except for the VAS and WPI. Measures were taken to limit missing data. That is, during data collection, each participant’s questionnaires were checked for completeness, and, in case of incomplete responses, the participant was encouraged to complete the missing questions. To ascertain valid data processing, all data were entered in two different numeric files by two blinded researchers and were later checked for discrepancies.

### 2.3. Central Sensitization Inventory

The CSI is a two-part questionnaire, of which part A measures a full array of 25 somatic and emotional symptoms associated with CS, scored with a 5-point Likert scale from 0 (never) to 4 (always), resulting in a total possible score of 100. Higher scores denote a higher degree of self-reported CS-related symptomatology. Part B asks if the patient has been previously diagnosed with seven CSS and three CSS-associated diagnoses, but it was not considered in our study. It was shown that a bifactor model containing one general factor representing “CS-related symptoms” and four orthogonal factors of “physical symptoms,” “emotional distress,” “headache/jaw symptoms” and “urological symptoms” fits the CSI structure the best [21,22]. The CSI has been found to be psychometrically sound in all published studies to date [13,20,26].

### 2.4. Short Form of the CSI

The CSI-9 was developed using Rasch Analysis [15]. It measures 9 somatic and emotional symptoms associated with CS, with a scoring similar to the CSI and a total possible score of 36. Higher scores denote a higher degree of self-reported CS-related symptomatology. The CSI-9 has shown acceptable psychometric properties and adequately comprises the factors identified in the original CSI [15].

### 2.5. Visual Analogue Scale

The VAS is a non-specific psychometric response scale used to measure the mean pain intensity experienced by the patient. The patient answers by marking a 100 mm line, where 0 mm is equivalent to “no pain” and 100 mm equals “worst imaginable pain”. The distance measured from the “no pain” end to the patient’s mark is the VAS score. The VAS is a valid and reliable tool to assess pain in cancer patients [27].

### 2.6. Widespread Pain Index

The WPI or formerly the “Regional Pain Scale” consists of a body diagram divided into 19 regions [28]. Patients are asked to indicate in which of these body areas they had pain during the last week, which gives a total score out of 19. The WPI is now widely used in the assessment of chronic pain patients [29].

### 2.7. Hospital Anxiety and Depression Scale

The HADS is a self-assessment scale developed to detect levels of anxiety and depression [30]. The HADS is a 14-item questionnaire that consists of two subscales, comprising 7 items each, to assess anxiety and depression, respectively. Each item is scored from 0 to 3 with a summary score between 0 and 21 for each subscale. Scores of 11 and above indicate the probable presence of a mood disorder. The HADS is a valid and reliable tool [31].

### 2.8. European Organization for Research and Treatment of Cancer QLQ-C30 (Version 3)

The EORTC is a questionnaire with 30 items scored with a 4-point Likert scale [32]. It was developed to assess the multidimensionality of the health-related quality of life of cancer patients. The EORTC incorporates one global health status scale, five functional scales (physical, role, emotional, cognitive, social) and nine symptom scales (fatigue, nausea and vomiting, pain, dyspnea, insomnia, appetite loss, constipation, diarrhea, financial difficulties). Higher global health status, functional scale and symptom scores indicate a higher quality of life, level of function and level of symptomatology, respectively. In this study, the nausea and vomiting, appetite loss, constipation, dyspnea, diarrhea and financial difficulties subscales were not used. It is a valid and reliable tool to assess quality of life in cancer patients [33].

### 2.9. Social Demographic Questionnaire

The social demographic questionnaire collected the following information: age in years, cancer type with diagnosis date, date of end of medical treatment, cancer treatment received among a list, comorbidities and current medication.

### 2.10. CSI Cluster Calculator

The CSI-CC is a tool to cluster patients into CS-related symptom severity subgroups. This tool has been developed in a large pooled multicountry sample of chronic pain patients and healthy subjects by hierarchical cluster analysis and latent profile analysis [19]. The CSI-CC uses the patients’ answers to the CSI to classify them in one of the three clusters labelled as follows: low level of CS-related symptom severity, medium level of CS-related symptom severity and high level of CS-related symptom severity.

### 2.11. Statistical Analyses

All statistical analyses were performed using the SPSS version 25 for Mac (IBM Corp., Armonk, NY, USA). Data distribution was visually inspected by Q–Q plots and the Kolmogorov–Smirnov test. Associations were calculated with Pearson correlation coefficient for parametric variables and Spearman’s correlation coefficients for nonparametric variables. If the correlation coefficient was >0.9, this was considered as a very strong association, between 0.7 and 0.9 was a strong association, 0.5 and 0.7 was a moderate association, between 0.3 and 0.5 was a weak association and <0.3 was a negligible association. The Cronbach α was used to measure internal consistency. An α between 0.50 and 0.60 was considered poor, between 0.60 and 0.70 was considered acceptable, between 0.70 and 0.90 was considered good and higher than 0.90 was considered excellent.

To examine the differences between the three cluster subgroups on the VAS, WPI and EORTC global health, ANOVA or Kruskal–Wallis tests were applied, with Bonferroni correction. The alpha for Bonferroni correction for multiple comparisons was set at 0.016 to control for the family wise error rate. Boxplots were made to compare the CS-related symptom severity clusters for the remaining measured variables. Patient responses were inspected for missing data. Statistical significance was set at *p* < 0.05 for all other tests.

## 3. Results

Data collection took place from November 2015 until March 2016. The present exploratory cross-sectional multi-center study included 69 BCS and 37 female healthy volunteers. Data of 65 BCS were used for analysis, because four were excluded before analysis: three because of metastasis and one because of pain unrelated to cancer. Data were missing for the subscale EORTC insomnia for only one patient, who was excluded for the corresponding analysis. For the other subscales, no missing data were present. The participants characteristics of the study sample are presented in Table 1.

Cronbach’s α coefficient for internal consistency was 0.921 for the full CSI and 0.860 for the CSI-9. All associations for the CSI and CSI-9 are presented in Table 2. Statistical analysis showed a very strong association between the CSI and CSI-9 (r = 0.92; *p* < 0.01). A strong association between the CSI and EORTC emotional (r_s_ = −0.72; *p* < 0.01) was found as well. A moderate association between the CSI and VAS (r_s_ = 0.60; *p* < 0.01), WPI (r_s_ = 0.62; *p* < 0.01), HADS anxiety (r_s_ = 0.68; *p* < 0.01), HADS depression (r_s_ = 0.67; *p* < 0.01), EORTC physical (r_s_ = −0.56; *p* < 0.01), role (r_s_ = −0.60; *p* < 0.01), cognitive (r_s_ = −0.66; *p* < 0.01), social (r_s_ = −0.51; *p* < 0.01), fatigue (r_s_ = 0.68; *p* < 0.01), pain (r_s_ = 0.61; *p* < 0.01), insomnia (r_s_ = 0.59; *p* < 0.01) and global health status (r_s_ = −0.55; *p* < 0.01) was found. The CSI-9 correlations did not drop by more than 0.10 from the CSI correlations, except for HADS anxiety (r_s_ = 0.57; *p* < 0.01), EORTC physical (r_s_ = −0.41; *p* < 0.01) and EORTC fatigue (r_s_ = 0.57; *p* < 0.01).

### 3.1. Clustering

Descriptive statistics for the three clusters of CS-related symptom severity are presented in Table 3. The low level of the CS-related symptom severity cluster was mainly composed of healthy volunteers (*n* = 33, 63.5%), whereas female healthy volunteers were a minority in the medium level of the CS-related symptom severity cluster (*n* = 4, 11.5%). No female healthy volunteers were found in the high level of the CS related symptomatology cluster.

The differences between the three cluster subgroups on the VAS, WPI and EORTC global health are presented in Table 4. The Kruskal–Wallis H test showed that there was a statistically significant difference in VAS score between the different CS-related symptom severity clusters (Kruskal–Wallis H = 43.90; *p* < 0.001, dl = 2). The low cluster statistically differed from the medium (Ranksum = −27.20; *p* < 0.001) and high (Ranksum = −49.91; *p* < 0.001) cluster, but the medium and high groups did not differ from each other (Ranksum = −22.71; *p* = 0.027).

For the WPI scores, the Kruskal–Wallis H test showed that there was a statistically significant difference between the different CS-related symptom severity clusters (Kruskal–Wallis H = 48.86; *p* < 0.001, dl = 2). The low cluster statistically differed from the medium (Ranksum = −28.81; *p* < 0.001) and high (Ranksum = −52.90; *p* < 0.001) cluster, but the medium and high groups did not differ from each other (Ranksum = −24.10; *p* = 0.018).

Furthermore, the Kruskal–Wallis H test showed that there was a statistically significant difference in EORTC Global health status scores between the different CS-related symptom severity clusters (Kruskal–Wallis H = 36.46; *p* < 0.001, dl = 2). The low cluster statistically differed from the medium (Ranksum = 21.27; *p* < 0.002) and high (Ranksum = 49.162; *p* < 0.001) cluster. The medium and high groups also statistically differed from each other (Ranksum = 27.89; *p* = 0.005).

### 3.2. Boxplot Description

Boxplots are presented and described in Supplementary File S1.

## 4. Discussion

Our results regarding the measurements properties of the CSI are in line with previous studies in non-cancer chronic pain populations [20]. The CSI showed moderate to strong correlation with the HADS anxiety (r_s_ = 0.68), depression (r_s_ = 0.67) and EORTC emotional (r_s_ = 0.72), which is consistent with the factor “Emotional distress” and previous studies [20,34,35,36,37,38]. Van Wilgen et al. reported that psychological factors and widespread pain contribute to the majority of the CSI variance in non-cancer chronic pain patients [34]. A moderate correlation between the CSI and EORTC cognitive was also highlighted (r_s_ = −0.66), which is consistent with the items 13 and 23 and the general association between pain and cognitive difficulties.

The WPI has consistently shown moderate correlations with the CSI in all studies to date, and the present study is the first to show this in BCS (r_s_ = 0.62) [34,36,39]. The relationship between CS and widespread pain has been strongly emphasized previously [11,40]. On the other hand, associations between the CSI and pain ratings have been less consistent in non-cancer chronic pain patients, and range from 0.19 to 0.60 in BCS [15,34,35,36,38,41]. This variability may be because of differences in sample sizes and populations. Moderate associations were found between CSI outcome and the EORTC physical (r_s_ = −0.56) and role (r_s_ = −0.60). Several authors have reported moderate correlations between the CSI and physical functioning measures [22,38,41]. The CSI yielded moderate correlations with the EORTC fatigue (r_s_ = 0.68) and insomnia (r_s_ = 0.59), which are symptoms commonly associated with CS [17,35]. Again, these results taken together relate to the factors “Physical symptoms” and “Headache/Jaw symptoms” of the CSI.

A moderate association (r_s_ = −0.51) between the CSI and EORTC social was found in this sample of BCS, whereas a priori, a weak association was hypothesized. This is remarkable as items measuring this social component are not included in the CSI. This suggest that symptoms assessed by the CSI might impact social functioning in BCS.

The convergent validity of the CSI and CSI-9 in BCS was satisfactory because the vast majority of our a priori formulated hypotheses were met: 22 out of 27 hypotheses (Table 3) [15].

The internal consistency of the CSI was excellent (α = 0.92), which is in line with previous reports that found values ranging from 0.87 to 0.91 [20,22]. A recent study evaluating the CSI in BCS found that an exploratory factor analysis yielded a one factor structure, which indicates that the CSI items measure the same construct [22]. Furthermore, a high internal consistency usually indicates that there are too much identical or comparable items, which supports the development of the CSI-9. As for the CSI-9, its internal consistency is also good (α = 0.86), which aligns with the original development version in non-cancer chronic pain patients [15]. In contrast, the fit of a unidimensional model for the CSI-9 might be questionable. Nishighami et al. statistical analysis suggested the presence of a second factor [15]. Despite the CSI-9 including items of the CSI, its dimensionality should be further explored.

Although often presented as such, the CSI does not reflect a direct measure of CS, but is a measure of general physical and emotional distress originating from symptoms attributed to CS. This is further supported by the moderate association observed between the CSI and global health status (r_s_ = −0.55), absence or negligible associations with quantitative sensory testing and the moderate to strong association with psychological distress [35,37,41,42]. To date, quantitative sensory testing is considered as the most objective measure of CS [43]. The CSI should not be used as a diagnostic tool for the same reasons and because of the high risk of false positives when using its cut-off score of 40 [18]. Nonetheless, considering its good validity and reliability, the CSI should be considered as a tool to quantify physical symptom severity and emotional distress associated with CS and CSS clinical presentation in BCS.

Finally, the limitations of the CSI arise from the lack of agreement about the definition of CS. While some authors consider it as a clinical picture, a diagnosis or a set of changes in the central nervous system, others advocate to reserve the term CS for the neurophysiological phenomenon related to its discovery [8,14,40,44]. To accurately measure a specific construct, it should be well defined and delimited [25].

Regarding the CSI-CC, the goal was to explore the extent to which the CSI cluster classification correlates with patient-reported outcomes [19]. In this study of BCS, for both VAS score and WPI, the low cluster statistically differed from the medium and high cluster, but the medium and high groups did not differ from each other. For the EORTC Global health status score, all clusters statistically differed from each other. Along with the trends observed on boxplots (see Supplementary File S1), the CSI-CC might be a useful tool to have a patient’s overall condition snapshot. Our results warrant further investigation in other samples. Moreover, studies evaluating the responsiveness and measurement error of the CSI in BCS and other cancer survivor populations would be beneficial.

### Study Limitations and Strengths

The small sample size and the broad definition of CS were the main limitations of the study. Another limitation was that the CSI-9 answers were extracted from the CSI, which might have impacted CSI-9 scores. Study strengths include using COSMIN guidelines to validate this tool. Additionally, the CSI was evaluated in clinical setting during regular rehabilitation in several private practices. Furthermore, the use of validated and widely used measures along with adequate statistical tools are important study strengths.

## 5. Conclusions

In summary, the study findings support the internal consistency and convergent validity of the CSI in BCS, and the internal consistency and convergent validity of the CSI-9 in BCS. This study, therefore, complements the already existing body of evidence for the validity and reliability of the CSI in cancer and non-cancer chronic pain populations, which underlines the use of this self-reported instrument to have a BCS’ CS-related condition overview. However, the CSI responsiveness and interpretability should also be investigated in order to use it as a follow-up tool. The CSI-9 is still in its early validation phase and should be further evaluated, as well as compared to the CSI, especially with regards to structural validity and content validity.

## Figures and Tables

**Table 1:** Participant characteristics (*n* = 102).

Variables	Breast Cancer Survivors (*n* = 65)	Healthy Volunteers (*n* = 37)
Age (Mean, [SD])	58 [9.5]	57.6 [9.6]
Cancer treatment *		
Surgery (%)	61 (93.8)	
Chemotherapy (%)	56 (86.1)	
Radiotherapy (%)	38 (58.5)	
Hormonotherapy (%)	45 (59.2)	
Targeted therapy (%)	3 (4.6)	
Still under hormonotherapy (%)	29 (44.6)	
CSI (Mean, [SD])	31.5 [15.2]	13.3 [6.4]
CSI-9 (Mean, [SD])	15.8 [6.5]	7.8 [3.6]
Reporting pain (n, [%])	57 [87.7]	0 [0]
VAS (Median, [Q1–Q3])	34 [13.5–50]	0 [0–0]
WPI (Median, [Q1–Q3])	4 [2–6]	0 [0–0]
HADS Anxiety (Median, [Q1–Q3])	4 [2–6]	3 [1.5–5]
HADS Depression (Median, [Q1–Q3])	2 [1–4]	1 [0–1.5]
EORTC		
Physical (Median, [Q1–Q3])	80 [73.3–93.3]	100 [100–100]
Role (Median, [Q1–Q3])	66.7 [66.7–100]	100 [100–100]
Emotional (Median, [Q1–Q3])	83.3 [66.7–100]	100 [100–100]
Cognitive (Median, [Q1–Q3])	83.3 [66.7–100]	100 [100–100]
Social (Median, [Q1–Q3])	83.3 [66.7–100]	100 [100–100]
Fatigue (Median, [Q1–Q3])	22.2 [11.1–44.4]	0 [0–11.1]
Pain (Median, [Q1–Q3])	33.3 [0–33.3]	0 [0–0]
Insomnia (Median, [Q1–Q3])	33.3 [0–66.7]	0 [0–33.3]
Global health status (Median, [Q1–Q3])	75 [58.3–83.3]	100 [87.5–100]

SD = standard deviation; Q1 = 1st quartile; Q3 = 3rd quartile; CSI, Central Sensitization Inventory; CSI-9, Short-form of the CSI; VAS, Visual Analogue Scale; WPI, Widespread Pain Index; HADS, Hospital Anxiety and Depression Scale; EORTC, European Organization for Research and Treatment of Cancer questionnaire. * Some patients received more than one therapy. Frequencies represent the affirmative answers for each therapy and each percentage is computed on the total sample size of BCS. Note: for normally distributed data, means and SDs are presented. For non-normally distributed data, medians and Q1–Q3 are provided.

**Table 2:** Associations with CSI and CSI-9 (*n* = 65).

	CSI	CSI-9
CSI-9	**0.92 ^†^**	
VAS	**0.60**	**0.55**
WPI	**0.62**	**0.61**
HADS Anxiety	**0.68**	0.57
HADS Depression	**0.67**	**0.61**
EORTC		
Physical	**−0.56**	−0.41
Role	**−0.60**	**−0.56**
Emotional	−0.72	**−0.66**
Cognitive	**−0.66**	**−0.72**
Social	−0.51	**−0.52**
Fatigue	**0.68**	0.57
Pain	**0.61**	**0.61**
Insomnia	**0.59**	**0.61**
Global health status	**−0.55**	**−0.49**

CSI, Central Sensitization Inventory; CSI-9, Short-form of the CSI; VAS, Visual Analogue Scale; WPI, Widespread Pain Index; HADS, Hospital Anxiety and Depression Scale; EORTC, European Organization for Research and Treatment of Cancer questionnaire. ^†^ = Pearson’s correlation coefficient; all others are Spearman’s rank correlation coefficient (r_s_). All correlations were statistically significant with *p* < 0.01; confirmed hypotheses are in bold.

**Table 3:** Clusters characteristics (*n* = 102).

	Low Level of CS-Related Symptom Severity (*n* = 52)	Medium Level of CS Related Symptom Severity (*n* = 35)	High Level of CS Related Symptom Severity (*n* = 15)
Breast cancer survivors (%)	19 (36.5)	31 (88.5)	15 (100)
Age (Mean, [SD])	58.7 [10.1]	58.8 [9.2]	52.7 [5.6]
VAS (Median, [Q1–Q3])	0 [0–4.75]	26 [5–44]	50 [40–62]
WPI (Median, [Q1–Q3])	0 [0–1]	4 [2–6]	6 [6–13]
HADS anxiety (Median, [Q1–Q3])	3 [1–4]	4 [3–6]	8 [6–13]
HADS depression (Median, [Q1–Q3])	1 [0–2]	2 [1–3]	6 [4–8]
EORTC			
Physical (Median, [Q1–Q3])	100 [93.3–100]	86.7 [80–93.3]	73.3 [60–80]
Role (Median, [Q1–Q3])	100 [100–100]	66.7 [66.7–100]	66.7 [50–66.7]
Emotional (Median, [Q1–Q3])	100 [93.75–100]	83.3 [83.3–100]	58.3 [41.7–66.7]
Cognitive (Median, [Q1–Q3])	100 [87.5–100]	83.3 [66.7–100]	50 [33.3–66.7]
Social (Median, [Q1–Q3])	100 [100–100]	100 [66.7–100]	66.7 [50–83.3]
Fatigue (Median, [Q1–Q3])	0 [0–11.1]	22.2 [11.1–33.3]	55.5 [33.3–66.7]
Pain (Median, [Q1–Q3])	0 [0–0]	16.7 [0–22.2]	50 [33.3–66,7]
Insomnia (Median, [Q1–Q3])	0 [0–33.3]	33.3 [0–33.3]	66.7 [66.7–100]
Global health status (Median, [Q1–Q3])	95.8 [83.3–100]	83.3 [75–83.3]	58.3 [50–66.7]

SD = standard deviation; Q1 = 1st quartile; Q3 = 3rd quartile; CSI, Central Sensitization Inventory; CSI-9, Short-form of the CSI; VAS, Visual Analogue Scale; WPI, Widespread Pain Index; HADS, Hospital Anxiety and Depression Scale; EORTC, European Organization for Research and Treatment of Cancer questionnaire.

**Table 4:** Participant-reported clinical variables by CSI clusters.

Reported Variables (Scores Range)		Low Level of CS-Related Symptom Severity (*n* = 52) ^a^	Medium Level of CS Related Symptom Severity (*n* = 35) ^b^	High Level of CS Related Symptom Severity (*n* = 15) ^c^	Kruskal–Wallis H	*p*
BCS (%)		19 (36.5)	31 (88.5)	15 (100)	/	
VAS (0–10)	Median, [Q1–Q3]	0 [0–4.75] ^b,c^	26 [5–44] ^a^	50 [40–62] ^a^	43.90	<0.001
	Rank	34.83	62.03	84.73		
WPI (0–19)	Median, [Q1–Q3]	0 [0–1] ^b,c^	4 [2–6] ^a^	6 [6–13] ^a^	48.86	<0.001
	Rank	33.84	62.64	86.73		
EORTC Global health status (0–100)	Median, [Q1–Q3]	95.8 [83.3–100] ^b,c^	83.3 [75–83.3] ^a,c^	58.3 [50–66.7] ^a,b^	36.46	<0.001
	Rank	66.03	44.76	16.87		

SD = standard deviation; VAS, Visual Analogue Scale; WPI, Widespread Pain Index; EORTC, European Organization for Research and Treatment of Cancer questionnaire. Superscript letters indicate which groups significantly differed from each other’s.

## Data Availability

All data will be available under the correspondence e-mail address for 3 years following the publication after justifiable request.

## Supplementary Materials

The following are available online at https://www.mdpi.com/article/10.3390/clinpract11030076/s1, File S1: Boxplot description of CSI clusters.
