# Supplementary material for: Assessment of Central Sensitization in Breast Cancer Survivors: Convergent Validity and Use of the Central Sensitization Inventory (CSI) and Its Short-Form as a Clustering Tool
Source: Clin Pract. 2021 Sep 7;11(3):607–18. doi: 10.3390/clinpract11030076 (PMC8482070; doi:10.3390/clinpract11030076)
Supplement: Supplementary file 1 [file clinpract-11-00076-s001.zip › clinpract-1301196-supplementary.pdf]

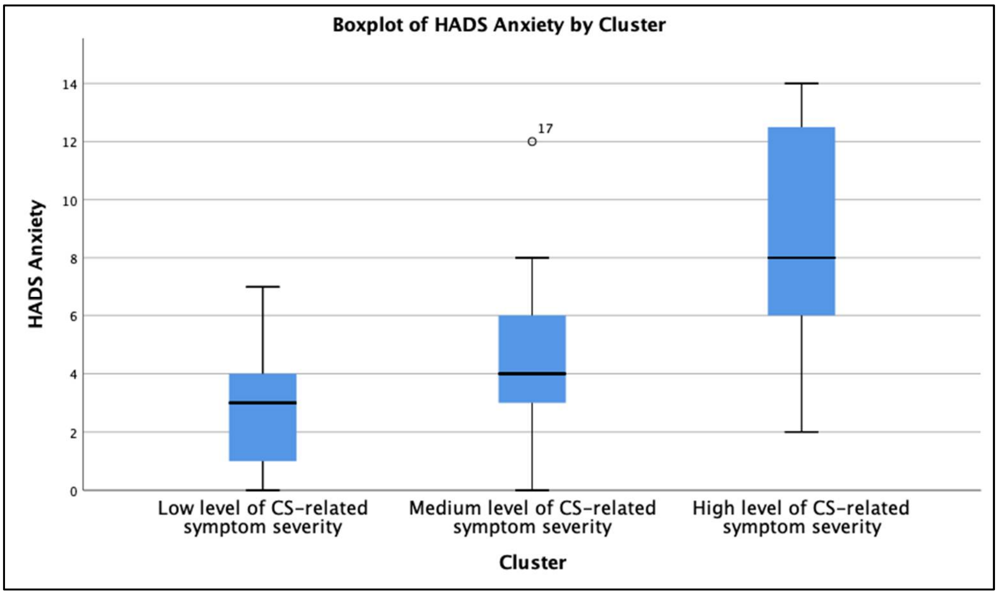

Figure S1. Boxplot showing HADS Anxiety score distribution by CSI cluster.

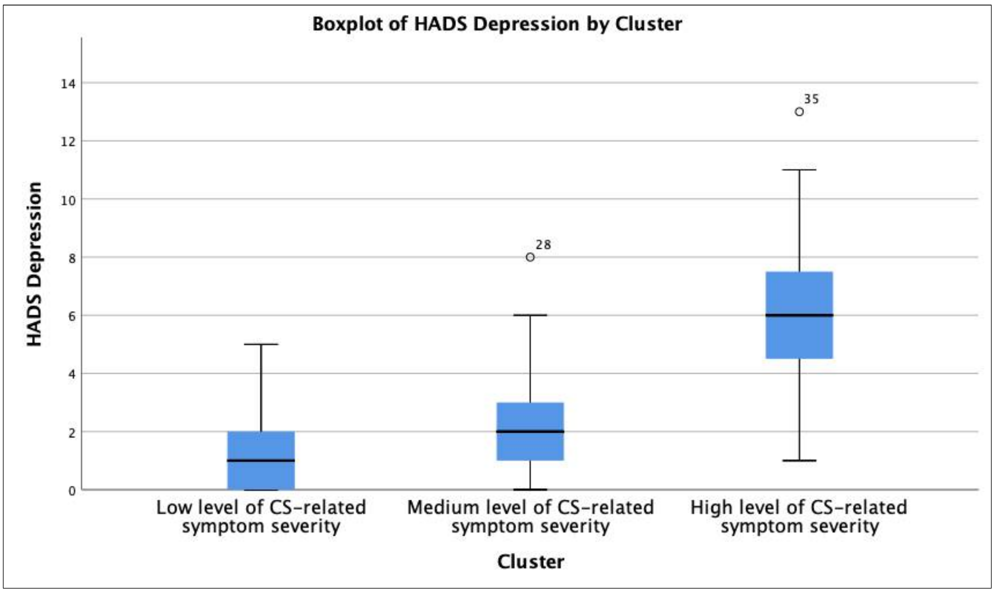

Figure S2. Boxplot showing HADS Depression score distribution by CSI cluster.

For both the HADS Anxiety (**Figure S1**) and HADS Depression (**Figure S2**), the low and medium cluster were comparable in terms of median and distribution around the median. For each scale, the high cluster median seemed to be noticeably higher and more variable than the two other clusters. For both the HADS anxiety and depression, BCS with a score indicative of a possible mood disorder (score ≥8) were almost found in the high cluster only.

In order to ease the EORTC subscales interpretation, the following rules were used. For the global health status and functional subscales (Role, Physical, Cognitive, Social): a score between 0 and 33.3 was considered as a functionality/Quality of Life strongly affected, between 33.3 and 66.6 as moderately affected, and between 66.6 and 100 as weakly affected or unaffected. For EORTC symptom subscales (Fatigue, Pain, Insomnia), a score between 0 and 33.3 was considered as low symptomatology or no symptomatology, between 33.3 and 66.6 as moderate symptomatology, and between 66.6 and 100 as severe symptomatology.

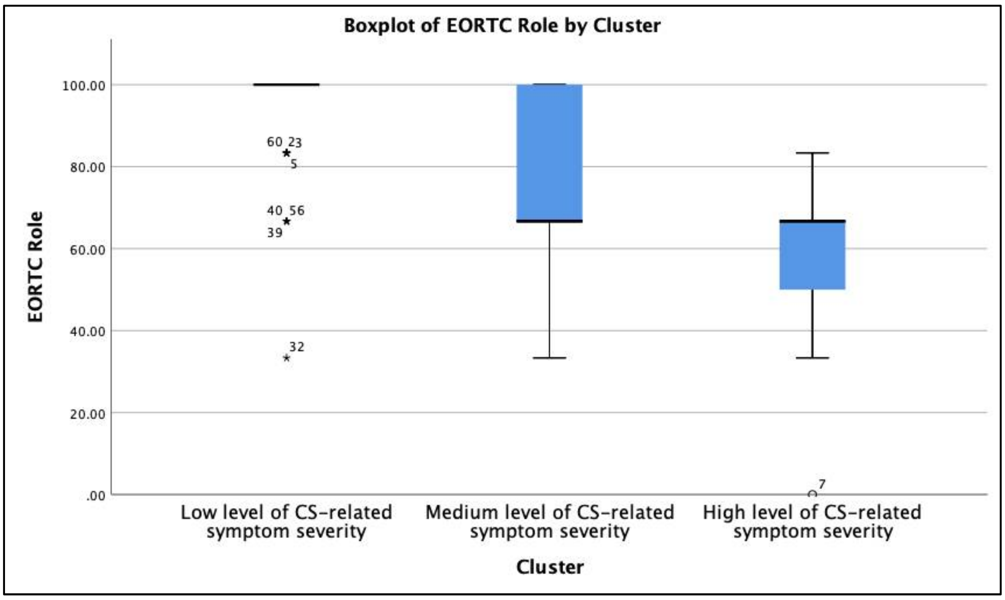

Figure S3. Boxplot showing EORTC Role score distribution by CSI cluster.

For the EORTC Role (**Figure S3**), the large majority of the low cluster reported unaffected function. On the other hand, half of the medium cluster reports moderately affected role function. The medium and high cluster had a similar median, but all patients of the high cluster reported weakly to moderately affected role function.

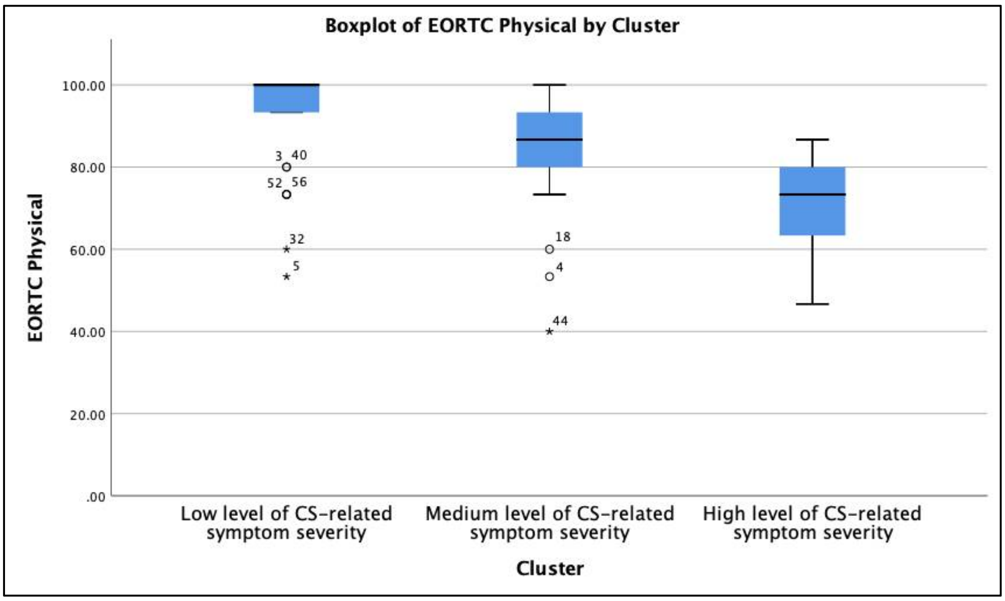

Figure S4. Boxplot showing EORTC Physical score distribution by CSI cluster.

For the EORTC Physical (**Figure S4**), we observed a stepwise decrease of median between clusters. At least 75% of the medium cluster reported a weakly affected physical function. All patients of the high cluster reported weakly to moderately affected physical function, with no patients reporting unaffected function.

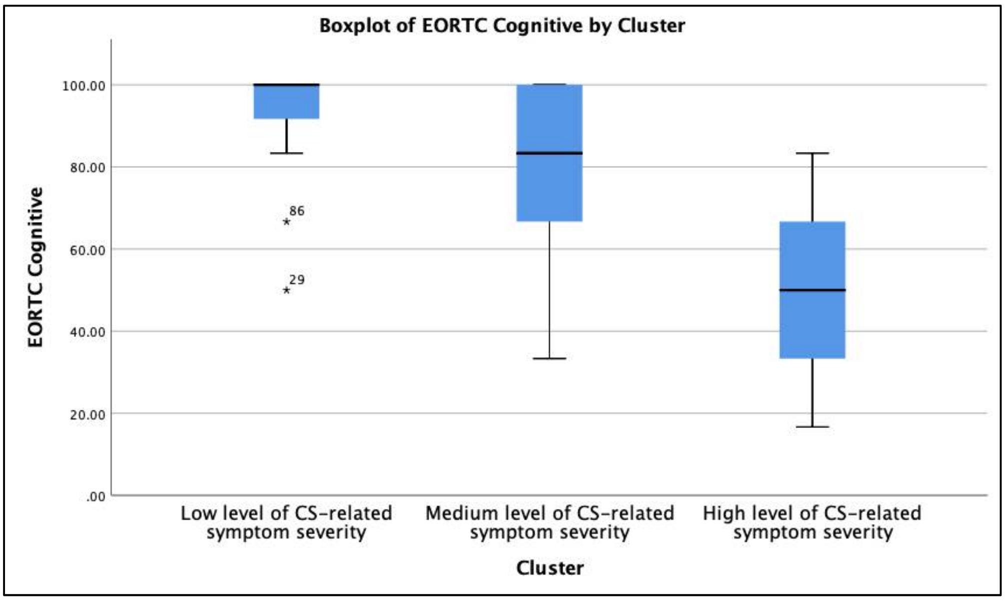

Figure S5. Boxplot showing EORTC Cognitive score distribution by CSI cluster.

For the EORTC Cognitive (**Figure S5**), we saw a stepwise decrease of median between clusters. The medium and high clusters had more variation than the low level cluster. At least 50% of the patients reported weakly to moderately affected cognitive function in the medium cluster. All patients of the high cluster reported weakly to strongly affected cognitive function.

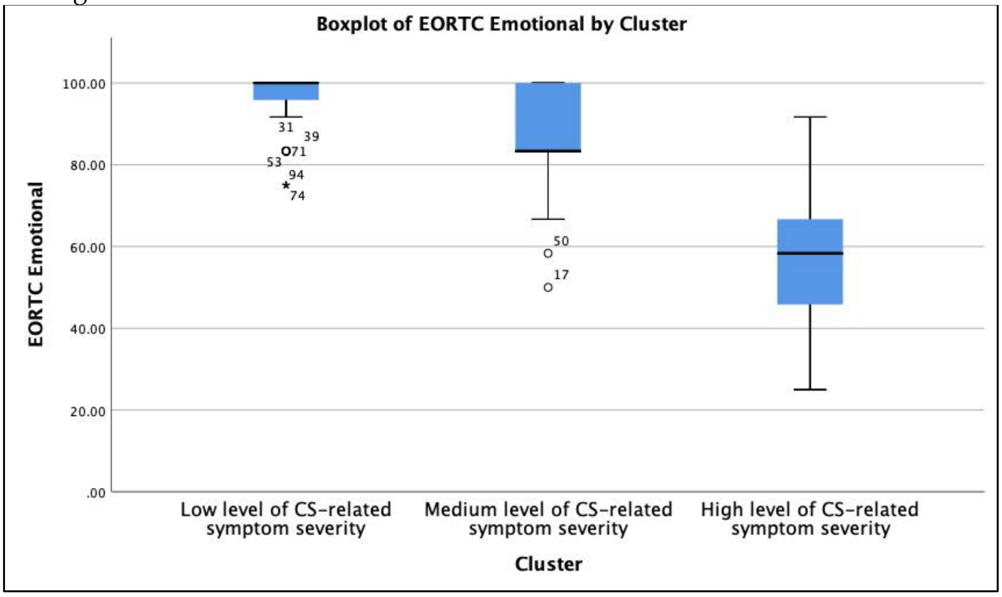

Figure S6. Boxplot showing EORTC Emotional score distribution by CSI cluster.

For the EORTC Emotional (**Figure S6**), we observed a stepwise decrease of median between clusters. The low cluster generally did not report affected emotional function. The majority of the medium cluster reported weakly affected emotional function. In the high cluster, all patients reported at least a weakly to strongly affected emotional function.

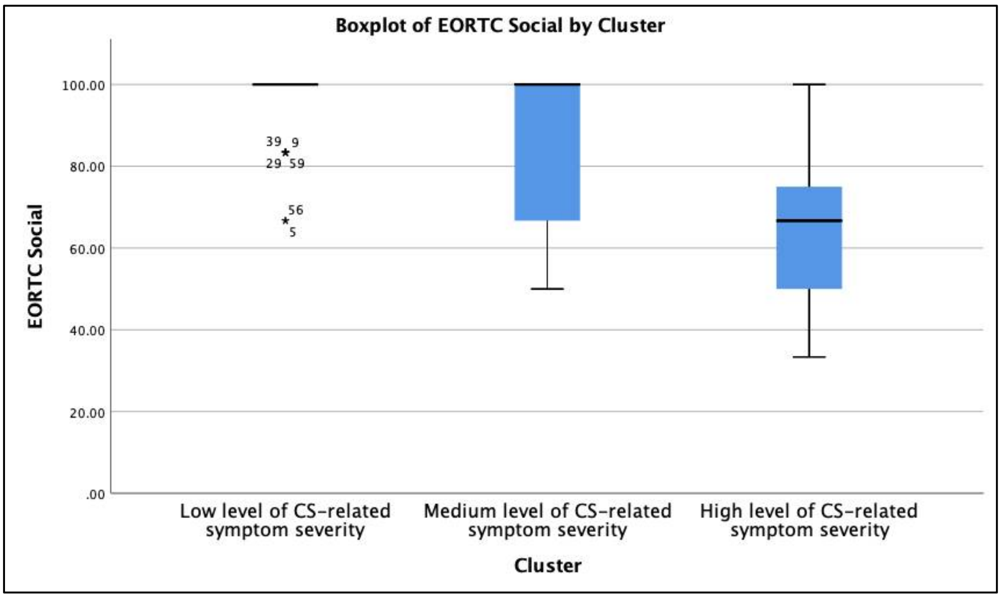

Figure S7. Boxplot showing EORTC Social score distribution by CSI cluster.

For the EORTC Social (**Figure S7**), the medians of the low and medium clusters were the same, however, at least one quarter of the medium cluster reported moderately affected social function. The high cluster median seemed to differ from the two other clusters, but the distribution was wide with patients reporting unaffected to strongly affected social function.

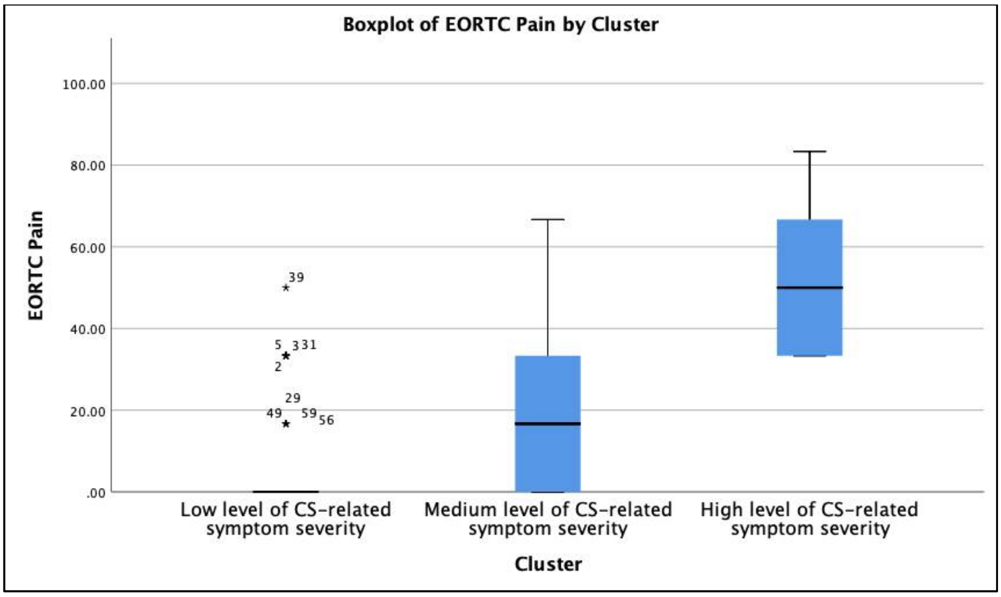

Figure S8. Boxplot showing EORTC Pain score distribution by CSI cluster.

For the EORTC Pain (**Figure S8**), we saw a stepwise increase of median between clusters. Participants in the low cluster generally reported no pain symptomatology. In the medium cluster, at least half of the participants were reporting low to moderate pain symptomatology. In the high cluster, patients were reporting moderate to severe pain symptomatology.

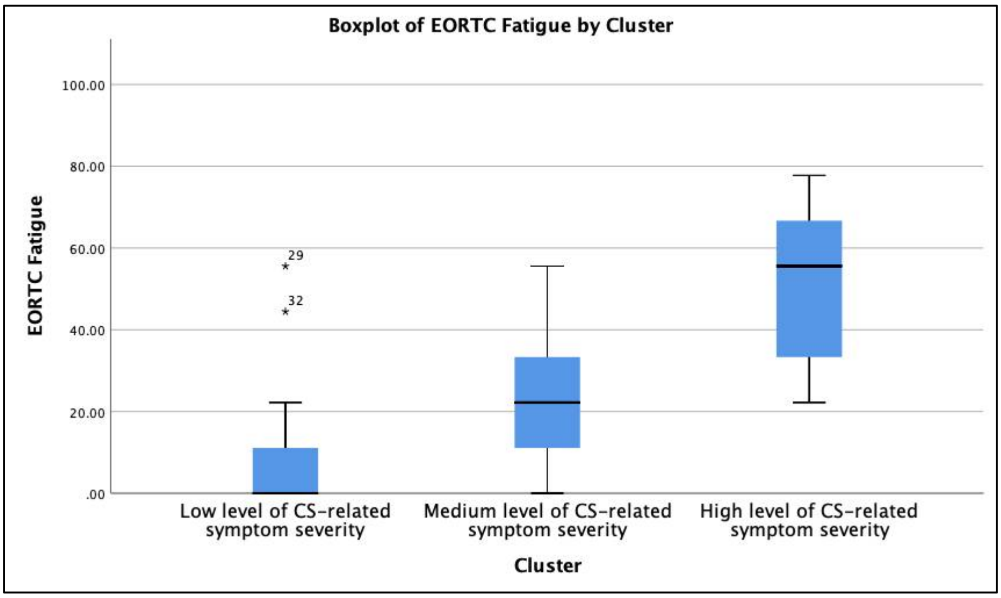

Figure S9. Boxplot showing EORTC Fatigue score distribution by CSI cluster.

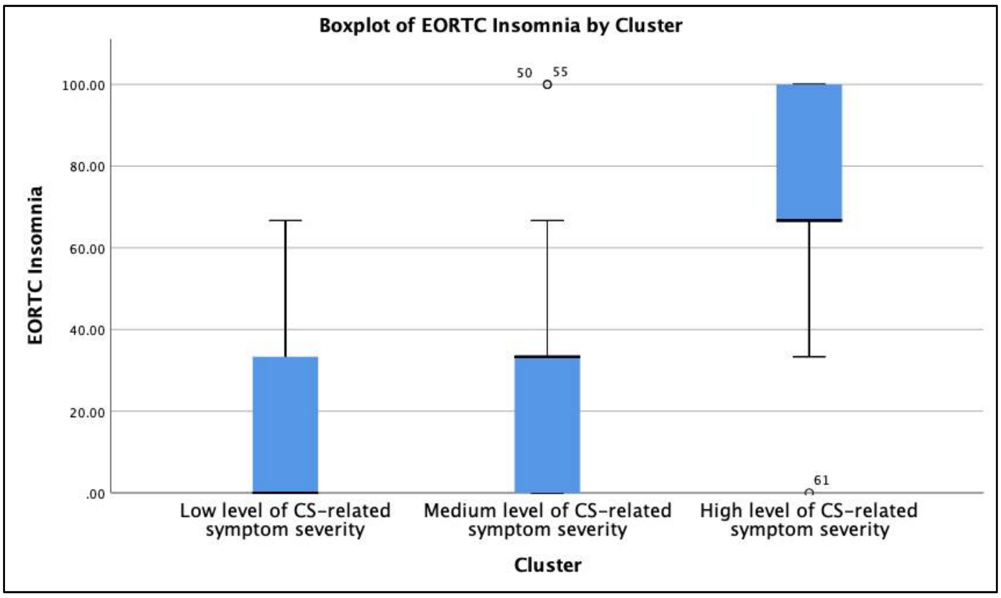

Figure S10. Boxplot showing EORTC Insomnia score distribution by CSI cluster.

For both the EORTC Fatigue and Insomnia (**Figures S9** and **S10**), we saw a stepwise increase in the median between the clusters. The score distribution around the median was wide. For the EORTC fatigue, all patients of the high cluster reported low to severe symptomatology. In the medium cluster, three quarters of the subjects were reporting low to moderate fatigue symptomatology. For the EORTC Insomnia, all patients in the high cluster reported moderate to severe insomnia symptomatology, except for one patient reporting no symptomatology. The majority of the subjects in the low and medium cluster reported low to moderate insomnia symptomatology.
